# Extra Supplement.—The Nursing Mirror

**Published:** 1893-12-09

**Authors:** 


"The Hospital" Nursing Mirror.

Being the Extra Nursing Supplement of "The Hospital" Newspaper.

[Contributions for this Supplement should be addressed to the Editor, The Hospital, 428, Strand, London, W.C., and should have the word "Nursing" plainly written in left-hand top corner of the envelope.]

### News from the Nursing World.

### THE NOVEL COMPETITION FOR NURSES.

Intending competitors are reminded that plans and descriptions of the Model Home for Nurses must reach Dr. Billings, Surgeon-General's Department, Washington D.C., U.S.A., not later than January 1st, 1894.

### CHRISTMAS FESTIVITIES.

Our friends are beginning to talk of "Christmas Entertainments," in fact at one hospital, at least, an early date is already fixed for the patients' fête. It is therefore time for us to remind our readers that due notice of festivities and descriptions intended for insertion in The Hospital must reach the office in good time. We are delighted to spare space in our columns for chronicling as many as possible of the entertainments given to patients and nurses, but naturally they are only suitable for the Christmas season, and we cannot permit them to exceed certain definite limits. It will therefore be of mutual advantage if all members of the large circle of our correspondents will aid us by sending in their contributions immediately after each event. Any approaching festivities of which preliminary announcements are desired must be officially reported to us ten days at least before they are to take place.

### THE LATE MISS ALLEN.

Over a hundred nurses have responded to the wish expressed by some of the pupils trained at the City of London Lying-in Hospital to raise a memorial to the late Matron, Miss S. E. Allen. The subscriptions have sufficed for the erection of a handsome marble cross with a suitable inscription at Highgate Cemetery, and also for the purchase of some engravings, with a memorial plate for the pupils' new dining hall. There is still place on the walls for several more pictures, and any old pupils who have not yet subscribed are invited to help in supplying them. Mr. Owthwaite will be glad to receive further donations.

### THE SCARCITY OF NURSES.

They are very short of nurses at the North-Western Hospital, with the result that the Committee report the present staff to be very much over-taxed. The medical officer has been empowered to engage a few nurses from some recognized institution at a guinea a-week, although several members of the Board expressed their doubts as to the wisdom of such a step. It will be interesting to know how many "recognised institutions" at present prove able to meet the demand of the Metropolitan Asylums Board. Not only do trained nurses usually get two or two and a-half guineas a-week for infectious cases, but the universal prevalence of many serious forms of illness is causing a serious strain on all nursing establishments. Many hospitals have difficulty in carrying on their ordinary work, owing to the presence of influenza amongst their staff.

### HARD LABOUR.

The carving and distributing of the patients' diets form important parts of a charge nurse's duty, and she seldom spares herself trouble in the matter. However, some institutions apparently expect much more than this from their staff, if we are to accept literally the following passage, which is copied verbatim from a correspondent's communication: "I must tell you that they require the nurse to be a cook, as all the cooking and weighing of the forty to fifty patients is done in the ward kitchen!" This savours of cannibalism, but possibly it is cooking "for," not "of" patients which is really in question.

### A SEASONABLE PLEA.

"They might come in usefully some day," is one formal excuse advanced in defence of hoarded garments. "They are too good to give away" is another and a worse one, and tempts the retort, "Are only valueless clothes fit for gifts?" In this bitter weather, with sickness and poverty everywhere, it is to be hoped that many will search through wardrobes and presses with an honest desire to see how much they can spare. When well-clothed folks are shivering in spite of food and fires, poor folks must suffer doubly. Tiny fires or none at all, insufficient food and limited garments (which do duty all the year round), and, to crown all, sickness. If ill enough for hospital, and lucky enough to find a bed, an invalid may be envied, for he lies in warmth and comfort with a cheery ward fire for a mental tonic. But the doctor's "You can go home to-morrow" comes all too soon for the individual patient, and the nurse's investigations as to "No. 7's," "own clothes," are so unsatisfactory that she looks round despairingly for something to add to them. "The patients' clothes are always on my mind in winter," remarked a ward sister the other day. "I declare I never see a visitor, whether man, woman, or child, in a new garment, without longing to ask if no half-worn things are ready to give away." Nurses, perhaps, more than any other workers are inclined to think unlimited hoarding partakes of the nature of a sin.

### HALF THE BATTLE.

"A good nurse is half the battle," said Lady Londonderry, speaking at a meeting at Silksworth, convened to discuss the establishment of a district nurse in this pit village. Stockton, Seaham Harbour and Seaham Colliery each possess a trained nurse who is highly valued by her patients, not only for the skilled care given to individual cases, but because she teaches the relatives how to tend the sufferers properly. Lady Londonderry's excellent speech was warmly applauded at the meeting, those present appearing to cordially endorse her opinion as to the need "for persons thoroughly understanding their business;" and again, "formerly it was thought that any woman could perform the duties of a nurse, whether she had any qualification or not, but now we realise that she requires as much training as is needed for any other purpose." All nurses owe thanks to Lady Londonderry for her able advocacy of the thorough training needed by all who aspire to fill the responsible position of district nurses. A cottage and an annual subscription of £25 form Lord Londonderry's contributions to the association. It is suggested that each man should pay a shilling and each lad sixpence, but this membership is to be entirely optional. At Seaham £50 was raised by the men and boys.

### A BASIS OF SELF HELP.

A committee has been formed in East Somerset for the purpose of establing a Cottage Nursing Association. It is to be worked on what is stated as a self-help basis. Cottagers subscribing two shillings annually, artisans three shillings, farmers five, and gentry ten, "and the same payments weekly when the nurse is in their houses." Supposing then that one of "the gentry" requires the services of this nurse for a long illness, it appears that he is permitted to claim them, paying only ten shillings instead of the thirty or forty which he could well afford to give to another, instead of leaving the cottagers to shift as best they can without the help to which they understood their small annual subscriptions had entitled them. The pay offered to the women competent to give what is said to be "good trained nursing," is at the rate of eight shillings a week for the first, and ten shillings during the second year, with uniform. The association is anxious to meet with irreproachable young women "with a turn for nursing," but does not say how much training is to be given.

### IN SOUTHERN SPAIN.

The Huelva Hospital, erected by the Rio Tinto Company, has an English matron and an English nurse, who are assisted by Spanish nurses. The hospital stands without the town, in its own grounds, and is in the midst of gardens and vineyards. The sanitary arrangements and the ventilation are admirably adapted to the south of Spain, and in the eight years which have elapsed since the erection of this hospital much useful work has been done, not only amongst the employés of the Rio Tinto Company, for whom it was primarily intended, but also for seamen visiting the port of Huelva. The latter are to be congratulated on the skilled care and nursing which this hospital gives to sick and injured sailors.

### TO PASS THE TIME.

"Did you enjoy being in the country, Tommie?" "Well, nurse, I ain't got nuthin' against the country, but I 'ates convalescing." Further inquiry elicited the fact that in Tommie's mind "convalescing" was synonymous with working. The stipulation that patients should be able "to do everything for themselves" when they exchange the quiet hospital ward for the bracing "home" did not disturb the lad just recovered from a long illness. However, when he got to the place which his imagination had idealised, he found quite a number of duties were expected of him both within doors and without. And then the other rules! There were so many of them, he confided to his old nurse. "It's werry well to give lazy chaps a few odd jobs to keep 'um amused, but I don't want no amusin', I don't. I'm at work all day when I'm well, and I wants to take it easy a bit now. So do the main o' the men. It's a reg'lar sell to shove a lot o' convalescing on to us poor weakly chaps, pretending it's just to 'pass the time' that we do all the work of the home," concluded Tommie, wrathfully. The lad's outspoken views deserve consideration, for certainly it is hard upon toiling breadwinners to be expected to do so much. If they get better air and food at a Home than they could obtain with their families, yet with the latter they benefit by the natural willingness to wait upon them. By his own people, however poor, small services are rarely expected from the man with crutches or a cough!

### WAITING FOR WORK.

If they are not fitted for any other career a great many people fancy themselves able to take up massage and to make a living out of it. There is every fear of this, like other branches of work, becoming thoroughly overdone. A number of trained hospital nurses are well-taught masseuses and get, doubtless, sufficient employment. But it is a pity for untrained girls with very small means at their disposal, to embark their all in taking "a few lessons," hoping to earn a comfortable income immediately. A good connection requires working up, for few doctors will be willing to entrust cases to a beginner, and therefore she may find long leisure weeks between occasional short cases which someone's good nature places in her hands. Would-be masseuses should count the cost before investing their small capital in instruction which, however good in itself, requires to be followed by an amount of steady practice during a long period when, perhaps, paid work has still to be waited for.

### SHORT ITEMS.

Funds have been raised to support a trained parish nurse at Leigh, Essex.—A small infirmary hut has been erected by the contractors of North Cornwall Railway. The Navvy Mission has loaned the furniture for it, and Canon Carter hopes to get a competent nurse. Six hundred navvies are working between Delabole and Wade Bridge, and their wives and families would no doubt be glad also to avail themselves of the services of a skilled nurse.—It is proposed to establish a village nurse at Greasborough.—The annual meeting of the Belfast Nurses' Home and Training School was a very satisfactory one, there being, for the first time for some years, a balance in hand. The sanitary defects within and without the house have been thoroughly remedied, the expenses thereof being defrayed by Mr. Forster Green.—Miss Emily Winifred Dickson is the first lady to become a Fellow of the Royal College of Surgeons of Ireland.—The Duchess of Teck has kindly sent to the Children's Hospital Home, Park Road, Kingston Hill, a large parcel of warm and useful clothing from the London Needlework Guild.—Miss E Barbara Harrison has been appointed Lecturer on Nursing to the Northampton County Council. She was trained at St. Marylebone Infirmary, and was afterwards Ward Sister there. Miss Harrison holds the L.O.S. diploma, and has had experience in district and fever nursing, and her future audiences are to be congratulated on the lecturer the County Council has secured for them.

### On the Nursing of Diseases of the Stomach.

### V.—DYSPEPSIA.

In a previous paper it has been shown that the presence of food in a healthy stomach leads to a flow of gastric juice and movements of the stomach of a twofold character—the churning and propulsive movements. Thus the food is in part digested in the stomach and then sent on into the intestine for the process to be completed. Indigestion, or dyspepsia, is any derangement of this normal process. Dyspepsia may be traced to: (1) the food; (2) diminution in quantity of gastric juice, or deficiency in either of its constituents, the pepsin or hydrochloric acid; (3) disorders of the movements of the stomach, either in the way of being excessive and hurrying on the food before it has been acted upon by the gastric juice, or in other cases being feeble or absent. With feebleness of the churning movements the food would not be brought into contact fully with the gastric juice. With feebleness of the propulsive movements, it would be delayed too long in the stomach, and its presence there would stop digestion, and probably fermentation of the contents would take place. In this process gases are given off which distend the stomach and cause flatulence and eructation. Though the causes of dyspepsia may be separated for the sake of convenience, it must be remembered that they are closely connected and generally associated together. As a rule, dyspepsia cannot be said to be due to one cause and one cause only, although primarily it may be either the food or the stomach that is at fault.

There are many ways in which the food may give rise to indigestion. The mastication of food may be incomplete, either as the result of undue haste in eating or from the bad condition of the teeth. Thus the food reaches the stomach in a condition unsuitable for the action of gastric juice. Insufficiency of food, and perhaps more frequently excess of food causes dyspepsia. Again, irregularity of meals, going long without food and then having a hearty meal in some persons gives rise to an attack of indigestion. Mental or bodily activity immediately before or after meals often interferes with digestion. Each person has to a certain extent to judge for himself what food he may take, for with regard to digestion there are many idiosyncracies which cannot be explained. But there are some substances which are more frequently than others sources of dyspepsia, especially new bread, potatoes, twice-cooked meats, shell-fish. Tea in excess should be mentioned, as perhaps it may be found to be a not uncommon cause of indigestion both among nurses and among many others who are working hard. Abuse of alcohol must not be omitted as a very common origin of dyspepsia. The effect of errors of diet, uncomplicated by any disease in the stomach itself, is seen in the case of children, who, either from the fault of those around them in not restraining them, or from their own fault, eat to excess of some unsuitable food, which is followed by an acute attack of dyspepsia, and this again by vomiting, which rapidly effects a cure.

In babies, again, "wind," giving rise to considerable pain and sickness is frequently caused by injudicious feeding. They may be fed too frequently, or at irregular times, whenever they cry, just "to keep them quiet," or if not breast fed they are in many cases fed on rusks, biscuits, &c., indeed anything but cow's milk properly diluted and sweetened. Cow's milk curdles in larger lumps than human milk, thus if not given with care it may disagree, and the mother, in consequence, ceases giving it to the child. But fresh milk given at regular intervals from a clean bottle, boat shaped, and not with an indiarubber tube, diluted with water, lime water, or barley water, either a half or a third, or less according to the age of the child, and sweetened with a lump of sugar, can generally be retained.

Deficiency in the constituents of the gastric juice and disorders of movements of the stomach will be considered together as due to either primary disease of the stomach or disorders in other parts of the body, reacting upon the stomach and thus interfering with its functions. Some of the more special diseases of the stomach will be considered later on; suffice it here to mention that dyspepsia would be a symptom of such diseases as gastric ulcer, cancer of the stomach, inflammation of the mucous membrane lining the stomach. Vomiting would often accompany the dyspepsia as mentioned in a previous paper. Dyspepsia is a symptom in many cases of disease of other organs. Thus in many cases of disease of the liver, the heart, or the lungs, the circulation of blood in the stomach is impeded, as has been explained, and its functions cannot be performed rightly. Again in anæmia, dyspepsia, with constipation, is a prominent symptom, and the dyspepsia is remedied by treating the anæmia and constipation. In fever the power of digestion is much lessened, the secretion of the gastric juice being diminished.

The actions of the stomach are influenced by disturbances in the nervous system, amongst others, by mental impressions, overwork, and emotions.

### Notes from Melbourne.

This is the twentieth year in which the Hospital Sunday Collection has been made in Melbourne. Owing to the universal depression a decrease in the donations was naturally anticipated on October 22nd, the day selected for receiving them; the churches returned £3,500 on this occasion, as against £5,392 last year. On Hospital Saturday only £100 was cleared, a great contrast to the £3,873, which was collected in 1888. The present mode of maintaining the charitable institutions by voluntary contributions, supplemented by a Government grant, has long been viewed with disfavour. During the past three years the amount of contributions has steadily grown less, owing to financial troubles, and this year, from the same cause, the Government has been compelled to reduce the charitable vote from £120,000 to £100,000. Thus every institution is in straits, and the present seems to be a suitable time to endeavour to obtain some alteration in the existing law. With this view a conference of representatives of people interested met in Melbourne in October. The meeting was unanimously agreed that the voluntary system had proved inadequate, but there was much difference of opinion as to what change was advisable. But the meeting as well as the Press and people of the colony were distinctly antagonistic to the idea of a poor-rate. The only feasible suggestion made was that the control of what were termed the absolutely necessary charities—such as general hospitals and benevolent asylums—should be vested in the municipalities. This to ensure economy, administration, and protection against imposition. The urgent necessity of placing the matter of maintenance on a sound basis was next day brought under the notice of the treasurer, Mr. Geoffrey Downes Carter, by a deputation of members of the conference. A promise was made to consider the matter carefully during the recess, regard being had to the recommendations of the Charities Commission, which lately presented its report, and that a Bill will be introduced into Parliament early next session. It is felt here, as well as in other parts of the world, that increased power should be given to the law, in order to compel people, who are able but not willing, to support their sick or poor relatives.

### Women's Work.

### FEMALE MEDICAL AID FOR THE WOMEN OF INDIA.

By Mrs. Scharlieb, M.D.

(*Read at the Women's Conference at Leeds.*)

### IV. EUROPEANS AND NATIVES.

Before the Victoria Hospital for Caste and Gosha Women was opened in Madras I visited native women in their own homes from six to ten or eleven every morning, and although I took all possible care of myself, driving in a closed carriage with a thick white quilted cover (in which I have seen the thermometer register 100 degrees), though I wore dark glasses and had an umbrella covered with white, yet I found the heat and the insanitary surroundings very trying, and have no doubt that house to house visitation under such circumstances of sun, heat, and glare, filthy accumulations of all kinds, and in small rooms sometimes destitute of any communication with the open air, would quickly incapacitate any European woman.

Other agencies for supplying female medical aid to the women of India are found in the several missionary bodies. Of these one of the best known is the "Zenana Bible and Medical Mission." This society supports several fully-qualified medical women from England, and trains native women as nurses and assistants. This is neither the time nor place to discuss the question of mixing medical and evangelistic work, but no one can overlook the great and good work done by the medical women sent by this society and other kindred societies to relieve the sufferings of the women of India.

Compared with these noble agencies private enterprise can do little, but must not be relinquished nor overlooked. Every fully-qualified medical woman who works among the women of India, and who does her work conscientiously and well, is a power for good. The testimony of Lord Roberts, recently Commander-in-Chief in India, is very noteworthy. He says, "The large majority of the women of India live outside these limited areas (the large towns), and for these, probably not less than one hundred and forty millions, skilled medical aid is at present an impossibility"—and he speaks of all we have yet been able to do as a mere drop in the ocean of Indian female suffering—"if they (the medical women) were multiplied by hundreds, or even thousands, their number would still fall short of what is really required."

The late Dewan to the Nizam of Hyderabad, the astute and far-seeing Sir Salar Jung, writing in 1880, said he was of opinion that it would be a benefit to India, which could not be exaggerated, if a fully-qualified woman trained in England could undertake medical work in each of the great towns of India. He estimated the number immediately needed at 1,025, but thought that number would be found insufficient. He also advocated two or more grades of medical women, so as to secure large numbers of less costly and more acclimatised workers.

As in the promotion of all large philanthropic measures, we earnestly need funds and workers—the one is useless without the other.

While we are slowly educating workers and collecting funds for thousands, nay, for millions of women, in the pathetic language of Lord Roberts, "skilled medical aid is at present an impossibility." Surely this unrelieved suffering is a national responsibility which each of us must be eager to share. Some can give their silver and gold, some can dedicate their daughters to this service, and some, happiest of all, can give themselves to the work. Let them realise, as I have had to realise, that "the work is great, the reward also is great, and the matter presseth. It is not incumbent on thee to finish thy work, but thou must not therefore cease from it." None of us will finish the work. It is too great. It extends from the snowy peaks to the southern waves, and it will last so long as there are women who suffer, and women to heal their sufferings. Each worker has but a span of time, a brief opportunity, a failing strength; each must work whilst it is called to-day, for the night cometh, and cometh quickly, but by earnest work among the sick, by patient teaching of future workers, the torch of truth must be handed on, and the work which cannot be completed must be continued.

### Nursing in Switzerland.

By Dr. Charles Kraft, Director.

### PRINCIPLES OF LA SOURCE.—IV.

### Internes and Externes.

The school is open not only to all applicants of the French tongue, but to every one, whatever her nationality, who is capable of speaking and writing French legibly. La Source admits indifferently, we repeat here, unmarried, married women, and widows. It receives externes and internes.

Internes.—Conditions: To belong to the Protestant Church, to possess certificates of health and morals, and to have a steadfast intention of carrying on the work of a nurse. The Director admits, of the applicants, those who present the best testimonials and appear the most capable of worthily fulfilling their duties, and from among these he chooses the ones who, having the least means pecuniarily, need to support themselves while consecrating themselves to the service of the sick. The lectures, board and lodging, light and fires are provided. Laundry work, and all the expenses of dress, are paid by the pupils. The internes live in the buildings, and take their meals there, under the supervision of the Director, the Directress, or Madame Krafft, who takes the place of the latter during her absence.

Externes.—The externes are selected from the lecture class, among those who wish to employ some part of their time in the gratuitous care of the sick, or those who for any reason, such as difference of religion, delicate health, or special duties, are prevented from taking the whole course and fulfilling its duties.

There is the same instruction for the externes as for the internes. The former are, however, released from a part of the practical work. The teaching is not free to the externes. Each one pays 120 francs for the complete course of lectures. In special cases the Director may, at his judgment, remit all or a part of the fixed price. (Several pensions and families living in the neighbourhood of La Source take externes to board at very moderate prices.)

### Pupils Graduated from La Source from 1859 to 1892.

For want of the early records and correspondence, it is unfortunately not possible to furnish accurate statistics of the pupils who attended the school during the first four years of its existence. After this period we find the following records:—

From 1863 to the end of September, 1892, 603 pupil nurses, 492 internes, and 111 externes graduated from La Source. Among these 519 were unmarried; 84 were married or widows. The number of regular pupils, taking the whole course of five months, has not varied greatly from the usual course of five months, has not varied greatly from the usual average—16 a year. Sometimes the number rose to 18, rarely to 20. The externes, who were not arranged for at the outset, did not form a part of the school during the first years. The school, at that time little known, did not gather without difficulty the 16 applicants that she had undertaken to train. By the goodness of God, however, all difficulties disappeared, and the school was enabled to carry out her programme. During the first 22 years (up to 1870) only two externes applied at La Source. From 1870 to 1881 the same number was received annually. At the present time, the applications each year are from 3 to 10, and we are now without any uncertainty on the score of recruits.

Nationality of the Pupils.

500 Swiss and 103 foreigners are distributed as follows:—

Swiss.—Vaud, 271; Berne, 89; Neuchatel, 53; Basle, 4; Argovie, 3; Saint-Gall, 3; Geneva, 18; Zurich, 16; Fribourg, 10; Valois, 3; Glaris, 1; Lucerne, 1; Thurgovie, 6; Appenzell, 4; Grisons, 5; and Soleure, 4.

Foreigners.—France, 55; Germany, 15; Great Britain 14; Russia, 7; Holland, 4; Denmark, 3; Belgium, 2; Italy 1; Australia, 1; Austria, 1.

Seventeen Cantons of Switzerland have sent us pupils. Five only—Uri, Schwytz, Unterwald, Zoug, Tessin—have no representatives in the school. To the Canton of Vaud, on the other hand, belong more than half of the Swiss pupils. Ten foreign nations have done us the honour to profit by the varied resources that the school offers. Among these France occupies the first rank, having sent us 55 pupils out of 103 that we owe to other countries than our own. The similarity of language partly explains this fact.

Work of the Six Hundred and Three Pupils of the School During and After their Time of Training.

We will not consider again the daily work of the pupils during the five months that they passed at La Source. A simple *résumé* of their visits and night duty will sufficiently illustrate. From 1883 to 1892 (that is, in 29 years), 58,189 visits and 4,932 nights of night duty with the sick in the town have been made by the pupils. These two totals give a mean of 2,006 visits and 169 nights of night duty yearly. Let us add to this sum the large one of the visits made and the watching done in the ambulance service, particularly at Pontoise (Lausanne) during the Franco-Prussian War (1871).

After graduates, a considerable number of pupils were appointed to positions by the Board. Others have found work for themselves in different countries, while some have given up their profession. The pupils are classified under the following heads: 1st. Those working in hospitals, public or private infirmaries, missionary nurses, 268. 2nd. Those who are gratuitously working among the poor, 92. 3rd. Those who can no longer pursue their work, or who for one reason and another have never pursued it, 59. 4th. Those whose address is unknown, 131. 5th. Those whose deaths have been learned, 53.

Our pupils may be found not only in Europe, but in Asia, in Africa, and in Australia. At home, the greatest number may be found by the borders of Leman, in Geneva, Vevey and Montreux. Lausanne has few; the other Vaudois villages still fewer. However, they are to be found in most of the Confederate Cantons, especially Berne and Neuchatel. In France—Paris, Lyons, Marseilles, Bordeaux, Nice, Cannes, Aix, Alois, Saint-Hippolyte, Montauban, and Annonay employ our nurses. In Italy they are working in Milan, Naples, Genoa, and San Remo. Ten or twelve of them are working in the two Americas. Some are missionaries; two in China, two in Africa, &c.

Let us now repeat, for the question of principle has the first rank with us, our pupils having finished their course are free—free to work where they will; free to remain in communication with the management of the school; free to enter the category of those classified "address unknown." We are satisfied to be able to give information concerning 472, or more than three-fourths of the whole number.

### Heroism.

A sunny face, though an aching heart;
A strong resolve when it's right to part;
Kind, friendly deeds, where no thanks are given;
When evil reigns, firm faith in heaven!

Nurse Beatrice Furnival.

### Causerie from New England.

### BOSTON, MASSACHUSETTS.

Two Famous Professors.

Harvard University has recently lost two professors, both of whom were devoted to scientific research. Herman August Hagen, professor of entomology, died November 9th in his seventy-seventh year. He was a Prussian, and a most interesting and picturesque figure in Cambridge circles. He was born in Königsberg, in whose famous University ancestors of his had held high positions for 250 years, so this family is a notable instance of the heredity of intellectual ability. In his early life he practised medicine in his native town, and was president of the City Council and member of the School Board. Louis Agassiz discerned his great capacity for scientific investigation, and in 1867 induced him to be his assistant in the Museum of Comparative Zoology at Cambridge, where he afterwards became professor. He published more than 400 scientific articles, but his most important work was the "Bibliotheca Entomologica," which gave him high rank among the scientists of the world.

David Scott Monecrieff, another Harvard professor, who began as a physician, and by a natural transition went into special scientific work, met a sad fate last August in Vingov, on the Saghalian Coast of Siberia. Word has just been received at the State Department from the Russian Minister at Tokio that Dr. Monecrieff started out for a sail, and as he never returned it is supposed that he was drowned. He was sent to Siberia by the Peabody Museum of Harvard University to pursue special studies in anthropology.

Improvements Required.

A year ago a committee was appointed by the Clinical Section of the Suffolk District Medical Society to secure improved sanitary legislation in Massachusetts. Three Bills were formulated. The first provides for advisory medical inspection throughout the State; the second for the registration of vital statistics under provision of the State Board of Health; the third makes obligatory a return of all contagious cases to the State Board of Health by local boards of health. Supported by the City and State Health Boards and the Mayor of Boston, and reinforced at the hearing by scientists from the Institute of Technology, the committee is greatly disappointed that the Legislature passed only the third Bill. These matters, of the highest sanitary importance, seem to have made little impression on the average legislator. But infectious diseases can never be stamped out until there is concerted action between local and State health boards, with a central national directing force at Washington.

No Progress Made.

The need of more stringent sanitary laws is constantly brought to notice. An outbreak of diphtheria in the western section of Lynn, Massachusetts, numbers thirty cases in one week only, seventeen of which developed in the Children's Home. The fact that nearly all the children in this home have either had the disease, or are now ill with it, indicates a violation of sanitary principles that is well nigh criminal. It is the old story over again. The authorities have traced the cause to defective sewers in the adjacent streets. If the law which the Suffolk Medical Society tried to secure for "advisory medical inspection" were in operation, the presence of homes for orphan and destitute children in streets which are death-traps, would be less likely to occur.

Mortality in Orphanages.

Nature seems to disapprove of orphan asylums and homes for destitute children, if the rate of mortality be the test, for it is asserted that the deaths sometimes rise to 200 and 300 per thousand. Experience, of the most costly and heart-rending character, has proved that children cannot be aggregated together with either physical or moral safety. Two wise women have evolved a system which has completely eliminated all asylums for children in Australia, which now boasts that in all her broad acres there is not a single orphanage.

The Australian Method.

Boston is investigating the Australian system, and Philadelphia has already begun to work on the same basis of finding a home and parents for every destitute or orphan child.

Each waif is taken to a receiving house until a permanent home in the country can be found. Each neighbourhood has a volunteer committee to canvass for available homes, and to report to the central board, great pains being taken to adapt the child to the home most suitable for its individual nature, and the foster parents receive $1.25 per week for its care. The school teachers, the police, the village health officer, and the committees all co-operate to see that the child is properly attended to. The child must be kept regularly at school until he is fourteen, then he is set to work at some trade, and his earnings placed in the Postal Savings Bank. At eighteen he goes out into the world a self-respecting, self-supporting citizen. At an expense of $10 annually the State has raised a boy or girl by this method who is not likely to become a pauper or a criminal. As civilisation advances the State must adjust itself to new methods, and orphans must no longer be left to churches or public charities.

### Nursing in the South Sea Islands.

(By Sister Victoria.)

In the Molucca islands of the Malay Archipelago, lying between Australia and Hong Kong, one scarcely expects to find much skilled nursing, nor does it exist, in this centre of the pearl fishery. On leaving Queensland and passing through the Torres Straits, one approaches these beautiful little islands, which are seen to the best advantage in the early spring, before the great heat. Even in February the sun had powerfully asserted himself, and 98 deg. in the shade was pronounced "a nice cool day!" Having visited many of these smaller islands, and found them much alike in the accommodation and treatment provided for the sick, it will be sufficient to describe one place only. The natives are of a poor physique; they are dark brown in colour, scantily clothed, and in general appearance and manners strongly influence a stranger to believe in Darwinian theories. Landing on the largest island, the so-called hospital is found to be a large white house, where a band of devoted Roman Catholic sisters attend on the sick.

As I carried an introduction, I was admitted without difficulty, and most kindly received by the Sisters, who appeared very willing to show their little institution. Clean, bare, and cool were the rooms occupied by the patients, the sun being carefully excluded in the usual tropical style.

The sick were evidently treated with the utmost leniency and kindness, but I must confess that actual nursing was quite a secondary consideration. One of the sisters informed me that surgery was only resorted to in cases of grave emergency, the disease most prevalent in the place being evidently fever.

Very decided interest was evinced in my uniform, which was carefully examined. After leaving the wards a great many questions were asked concerning hospital life and work in England, the first inquiry being, "Are all the hospital sisters in England vowed? If not, why do they call themselves sisters?"

They appeared amazed to hear that bodies of women could work harmoniously, although the individuals belonged to various sects; but they were chiefly surprised to learn that Roman Catholics were admitted to the same hospitals and even to the same wards as other nurses, and that differences in creed did not interfere with the good work done in English hospitals.

I told them that the example of the Roman Catholic sisters, in their unselfish devotion, constantly acted as a stimulus to other workers.

Then we discussed the question of ward maids. They could not understand nor sympathise with their position, for Sisters of Mercy do all the work of the hospital, including washing, cooking, and cleaning, as well as nursing, and most exquisite needlework, which they sell for the benefit of the institution.

I shall aways remember my visit to these gentle sisters, and their

Calm, hushed presence,
Bringing rest to those who felt and understood
The dignity of womanhood.

English is not spoken or understood, but Portuguese, Spanish, or French serve for conversation. I suggested sending some nursing papers out to them as one said she could read English although she could not speak it, and she promised to translate the contents to the others. Twice already I have received word of how much they appreciate learning through these journals. I asked one of the sisters if they were not lonely out there by themselves, but she immediately assured me of their happiness and contentment, and quoted those lovely lines:—

And for the happiness of which I spake,
I find it in submission to His will;
And calm trust in the Holy Trinity
Of Knowledge, Goodness, and Almighty Power.

The old Asiatic proverb says, "There is much time in an opportunity," but I did not find it so that day, four hours having passed away too quickly. As the bell sounded for Evening Service in the hospital I took my leave of these kindly women, who give up their lives to help the far away needy, whom they succour both in body and soul.

Returning to the ship, I pondered over what I had seen and heard in my day's experiences, and

So mix for ever with the past,
Like all good things on earth.

### Minor Appointments.

Grimsby and District Hospital.—Miss Alice Elce has been appointed Day Sister at this hospital. She was trained at the Royal Infirmary, Manchester, and worked there for six years; was afterwards charge nurse at Fir-Vale Infirmary, and at Burton-on-Trent Infirmary. We wish Miss Elce every success.

St. Mary Abbotts Infirmary, Kensington.—Mrs. Hopkins has been appointed Night Superintendent at this infirmary. Mrs. Hopkins was trained at Guy's Hospital, was for a time Matron at the East Grinstead Cottage Hospital, and for the past four years has been Charge Nurse of the enteric wards at the North-Western Hospital.

Salford Union Infirmary, Eccles.—Miss Frances Joy has been made Night Superintendent and Assistant Matron at this infirmary. She was trained at Crumpsall Infirmary, Manchester, did private nursing in Dublin, and was Staff Nurse at Burton-on-Trent Infirmary for two years. Miss Joy was afterwards Head Nurse at Clayton Hospital, Wakefield, and then held the post of Sister at Dr. Steven's Hospital, Dublin. Miss Joy has very good testimonials, and we wish her continued success in her future work.

### Wants and Workers.

Warm garments of all kinds are urgently wanted for the patients' Christmas gifts at the Guest Hospital, Dudley. Address the Matron.

Patients are received into the house of a lady living at Brighton, where they meet with every care and attention. Mental or infectious cases not received. Address R., care of Editor, The Hospital Office, 428, Strand.

Gifts are solicited for the patients' Christmas tree at the Lloyd Cottage Hospital, Bridlington Bay. All contributions will be gratefully received by Miss Hutton, the Matron.

### Everybody's Opinion.

[Correspondence on all subjects is invited, but we cannot in any way be responsible for the opinions expressed by our correspondents. No communications can be entertained if the name and address of the correspondent is not given, or unless one side of the paper only be written on.]

### WORKHOUSE INFIRMARIES.

"One Who Knows" writes: In a paragraph headed "At Last" in The Hospital of November 4th mention is made of "the strong prejudice against workhouse infirmaries by many trained nurses." Two explanations are suggested, neither of which is perhaps as weighty as the real facts. All persons employed by the guardians are called "officers"; the gate porter, the coal carrier, or dispensary messenger is as much an "officer" as the nurse or the matron. A free-and-easy familiarity exists, and where a newcomer attempts to treat those above her with respect, trusting to receive deference from those under her, she meets with insolence. For example, the head nurse reports a bath out of order. In a voluntary institution a workman would be promptly sent to attend to it, while in a workhouse infirmary the engineer comes in just when he chooses, and is probably impertinent if the work be reported not done after he has neglected instructions to do it. The gate porter is often another source of annoyance. Strict rules have to be kept of all who go in or out, and therefore he has many opportunities of being objectionable, and the whole tone is low amongst the men employed in these institutions. If food, always sufficient in quantity, is wasted through bad cooking the cook will suffer no complaints; she is an officer, and in many cases observes no allegiance to the matron, to whom she is barely civil. Any nurse trained where the matron is the acknowledged head of the female staff, and where all the men employed are civil and respectful, cannot but feel the difference in a workhouse infirmary. Again, there is to be considered the exceedingly trying chronic cases, with no special interest attaching to them from a nursing point of view, yet requiring constant attention. The great preponderance of such cases over acute ones causes an active-minded nurse to weary, and to draw comparisons with the successful results seen in other and more congenial fields. In spite of the increasing interest and the urgent need for trained nursing in workhouse infirmaries, a good class of women will not easily be obtained until some changes are made.

### IRISH DISTRESSED LADIES' FUND.

Lord Waterford, Chairman, London Committee Irish Distressed Ladies' Fund, writes: I hope you will allow me again to appeal to the public through your columns on behalf of the Irish Distressed Ladies' Fund. This charity is sadly in need of funds to keep it in existence, as this year the subscriptions have fallen off; and although I am obliged to make this application some time before Christmas in consequence of my having been ordered abroad, I hope the public will respond to this appeal in the same liberal manner they have done on previous occasions. The calls upon the charity have not decreased. During the present year to September 30th the London and Dublin Committees have expended the following sums: Pensions, £1,592 6s.; education, £208 13s. 3d.; allowances in cases of sickness, £87 2s. 6d.; other necessary grants, £386 8s. 6d.; making in all the large sum of £2,274 10s. 3d. This is exclusive of the expenses of administration, and although this seems to be a very large sum, yet it has been necessary to curtail the expenditure as far as possible, and many most deserving applicants have not received as much relief as the joint committees considered the cases required. If the public could see the desperate struggle for existence and the terrible trials bravely endured by those poor ladies who have been driven from their homes and left almost penniless upon the world by the agrarian agitation in Ireland, I feel certain that they would give some share of the charity which flows so largely at this time of the year to the fund. Each case is most thoroughly investigated, and great care is taken to give only to those really deserving, whose fortunes have been derived from Irish land, and who through no fault of their own have been left destitute. The object of this charity is to give work to those dependent on it, and thus encourage the ladies as far as possible to help themselves. This work is sold at the London and Dublin depôts of the charity, and the public would be giving grea assistance if they would buy from these depôts, where they can obtain clothing of the most excellent workmanship at a very moderate price. I hope you will allow me to say how grateful the Committees are to those who subscribed to the fund during the past year, and they sincerely trust that they may again be liberally supported with subscriptions in order that the good work which this charity has carried on for a number of years may not be stopped by want of funds. The Secretary will thankfully receive and acknowledge any subscriptions at the office of the charity, 17, North Audley Street, London, W., which is also the depôt for the sale of the Irish Distressed Ladies' work.

### ASYLUM v. HOSPITAL TRAINING.

An "Asylum Medical Superintendent" writes: I cordially agree with the views expressed by Dr. Harding in the November 25th issue of The Hospital, and consider he is worthy of all praise in his endeavour to improve the status of asylum nurses. I certainly think the time has come when the mental nurse must assert herself a little, and show the world that she is not one whit inferior to the hospital trained nurse. When asylum nurses, through the medium of your paper or otherwise, show those interested in their welfare that they themselves are anxious to have their status recognized, I am sure that the responsible heads of asylums will do all in their power to help them. I will be glad to hear if Dr. Harding has any practical suggestions to offer. The active interest which the Medico-Psychological Association now takes in the staff of asylums in granting them certificates after examination, written and oral, after two years' training, is, I think, an indication that the medical superintendents of asylums have the interest of their nurses at heart.

---

"Heron Hay" writes: Surely the thanks of all asylum nurses are due to Dr. Harding for his encouraging letter in The Hospital on November 25th. There is a decided feeling among such nurses that no one, not even medical superintendents, sympathises with them in the trials which their especial work daily brings. But on reading Dr. Harding's letter we can at once see that he at least is doing his best to place our work on a deservedly higher platform than it has as yet attained. I do admit that in many asylums women of an inferior class are admitted as attendants whom it would be an utter impossibility to train into good nurses, but is not this the fault of superintendents? If every superintendent made it understood that only women of a thoroughly good stamp would be admitted, very soon would those of an inferior class learn that it was useless for them to offer themselves as candidates for such responsible posts. Only then will the right women come willingly forward to this work. Berrywood is not the only asylum which gives a thorough training to its nurses, and on comparing the published examination papers set at Berrywood with the ones I have been accustomed to for the last three years I find there is nothing in any one of them that we have not been expected to know at the end of two years' training here. I think it would be a great injustice to many superintendents, and nurses, too, if an idea got abroad that Dr. Green's nurses were better trained than the nurses of many another asylum until such has been proved to be actually the case, but already we read that "fourteen public associations have undertaken to recognise and support the three years certificate about to be given by the Northampton County Asylum." This being the case, what is the use of the Medico-Psychological Association certificate which I hold?

### Where to Go.

Trained Nurses' Club, 12, Buckingham Street.—Lecture on Friday evening, December 15th, by Dr. F. J. Fielder, "On the Diseases of the Heart and Lungs."

### Experiences of a Lecturer in a Rural District.

(By an Experienced Speaker.)

There is certainly an amusing aspect as well as a deeply interesting side to the life of the teacher of hygiene and nursing under the County Council's technical education scheme. Arrived at a remote part of the kingdom—think of it, north or south, east or west, which ever you prefer, but always as a lovely, hilly country, my mission being to cottagers. The greater the ignorance the more slovenly their way of living, the more need of hygiene teachers; of this I was fully persuaded. There is not a more important movement on foot than that of teaching health and nursing in the rural districts. Many facts with which I have been face to face these last few months prove this.

First, there is the pitiful lack of knowledge as to the simplest rules of health and how to preserve it; the utter ignorance as to what to do till the doctor comes; how to prevent the spread of infectious diseases, &c. Secondly, the gratitude with which any teaching on these points is received. Thirdly, the help and relief the local doctors feel, where the people are taught, with some intelligence, to carry out his orders. Several very kind and able doctors have said to me, "If you would simply go round to the villages and teach the people how to make poultices and wring out fomentations, you would be doing a great work, and we should greatly welcome the effort." The doctors tell me they are constantly sent for, to outlying places, miles away; when they arrive, the thing they find necessary is a poultice; they order one. The woman of the house has not the remotest idea how it should be made, and the doctor, tired and hungry after a long round, has to stay to make and apply it himself.

I always make it my business first thing on arriving in a new centre of work to call on the doctor of the place. Excellent ladies have arranged the times and places of the lectures, but I feel strongly that the doctors know best the needs of the people, and I gladly take hints from them as to what teaching will be the most really and practically useful. They always receive me kindly and put me in the way of doing most good.

Perhaps one of the saddest aspects of ignorance is shown in the feeding of infants. I walked into a cottage a few weeks since, and there found two inhabitants—a baby and a pig. The baby was lying on the floor chewing a lump of bacon. It had finished its complement of bread. This child was aged four months. Of course, rickets and other such diseases abound, but as the mothers don't know the causes which bring about these complaints, it is no wonder that they give these babies things impossible to digest, and, therefore, the appalling statistics as to infant mortality are by no means surprising. This is a point which makes one feel strongly the need of teaching hygiene as well as nursing on the principle that "Prevention is better than cure." I was much struck the other day in a cottage hospital, when visiting a patient in whom I was interested, to see also a sweet-looking child in the ward visibly "ricketty." It was visiting day, and the child's own mother sat there nursing her. I ventured to ask if she knew what was the matter with the little girl. She said, "Weak legs." A question as to what she had fed the child on when it was but a few months old elicited the the answer, given with proud satisfaction, "Why, anything; bread, or potatoes, or bacon, or whatever we were having. You don't suppose I stint my children, do you? They have the same as we do"—and at four or five months old! Of course she was very sorry for the child to be now ailing, but it never dawned on her that the prevention of such illnesses lay in her own hands. In rural districts teaching hygiene specially embraces the pointing out of certain rules by which sickness may be avoided and health kept. The people seem greatly impressed, and readily jump to the conclusion that the speaker owns miraculous powers. For instance, I have several times been asked to charm away warts; and once an old woman stopped me in the village street and said, "My daughter has had constant fits these last thirteen years, and I thought perhaps you might just tell me some little thing that would stop them." Besides these amusing incidents from the people themselves, the organizers of the movement are sometimes unintentionally entertaining. People countenance lecturing in their districts from such different motives—some, because they really want their people helped; others, simply "to please the president"; some, because they never like to be outdone by other parishes in any movement on foot. Under the last two conditions a lecturer naturally meets with curious treatment occasionally. At one place I was greeted by "You see I felt bound to have these hygiene lectures, because Mrs. So-and-so asked me, and such-and-such a parish was having them, and I never like them to get the better of me. So here you are and we must make the best of you." (My spirits became decidedly low.) "Now not long since we had cooking classes, and at the end of them there was something to eat, so that made it worth while attending; but there will be nothing at the end of yours, and they are bound to be very dull." (My spirits went down lower still.) "Don't speak too long," my hostess continued, "for they will all be asleep in half an hour. Personally, I can't even think what hygiene means." Truly an encouraging greeting for the lecturer who had just arrived as a stranger, and was about to give the first of a course of four lectures. However, there was satisfaction in winning over that lady, and she afterwards owned that hygiene was full of interest, and would have liked each lecture "to go on for hours." But she who takes up health teaching must be prepared for many a little roughness; very off-hand treatment to begin with. If fully persuaded of the importance of her subject, if in full sympathy with the people to whom she speaks, the feeling that she has it in her power to be the real helper of the poor will generally cause the most prejudiced of hearers to give way. Then she will be shown much kindness, and will have the intense satisfaction of feeling her work has been of real benefit. It is worth while to wade through much preliminary discomforture and misunderstanding to accomplish in the end a mission most necessary and useful.

### Medico-Psychological Association of Great Britain and Ireland.

The following is a list of those who obtained the certificate of proficiency in nursing at the examinations held in November, 1893:—

City of Birmingham Asylum, Winson Green.—Females: A. A. Jones, G. Jones, S. Keen, and A. Truss.

County Asylum, Gloucester.—Male: E. Cordwell.

Bethlem Hospital, London.—Males: E. J. Cantle and A. Cantle. Females: E. C. Harrington and M. A. Lulham.

County Asylum, Rainhill, Lancashire.—Males: J. R. Bennett, A. Eggo, A. Gore, J. Hazlehurz, J. Kitchen, H. Morgan, T. Morton, J. Ross, A. Smith, W. H. Spencer, J. Smallshaw, J. L. Stanton, and W. H. Turner. Females: E. Angus, A. Brotherton, M. E. Bailey, M. Cheese, H. L. Eastmond, H. Day, B. I. Dovey, M. A. Freebury, C. E. A. Robinson, M. Randel, and E. Watkinson.

Holloway Sanatorium, Virginia Water.—Males: R. Bryant, J. W. Burgayne, T. McGann, T. S. Hunt, H. Laney, A. H. Wayne, and J. G. Stamp. Females: C. Plowright and K. Plowright.

Brook House, Upper Clapton.—Males: J. Dodkins, J. Eveleigh, T. G. Jennings, and T. Weatherley. Females: E. Barkwitz, C. A. Hobbs, A. M. Jones, A. Standfast, A. Sissette, M. H. Sweet, J. A. Smith, and A. Thacker.

Redlands, Tonbridge.—Female: M. D. Harmer.

Royal Asylum, Dundee.—Males: A. Galbraith and A. Soutar.

Derby County Asylum reported last week.

### The Book World for Women and Nurses.

[We invite Correspondence, Criticism, Enquiries, and Notes on Books likely to interest Women and Nurses. Address, Editor, The Hospital (Nurses' Book World), 428, Strand, W.C.]

Woman and the Man. By Robert Buchanan (Chatto and Windus, London, 1893.)

One might quite be forgiven if one observed that it requires the certification of Mr. Buchanan's name to suggest this work as being his authorship, for little in his latest novel bears out the high literary reputation which the writer has so deservedly established for himself. The plot of the novel under discussion is neither on an original theme nor is the interest engrossing. Even with the characters themselves one feels a certain familiarity, as if one had met them before in other costumes. The young and beautiful bread-earning wife is no novelty, either in fiction or, alas! in reality, any more than is her brandy-drinking, ill-matched mate. And yet, to be strictly just, it must be admitted in all fairness that there are certain situations depicted in the pages of "Woman and the Man" which undoubtedly do bear the stamp of originality, of indeed audacious originality. For instance, that chapter which in the first volume is headed "A Thunderclap" surely establishes a precedent of almost unequalled dramatic effect in the annals of any first-class literature. Here it is that the villain of the piece, the husband of the lady to whom we have referred, most regrettably returns home after an absence of many years, during which time he is supposed to be dead. The *soi-disant* widow at this point in the narrative one day enters her drawing-room only to discover the O'Mara, supposed to be deceased, playing "Home Sweet Home" on her piano. But that is not all. She comes into her house not alone, but is accompanied by a certain worthy baronet, whom only the previous day she had consented "with a gentle gravity" to marry forthwith. In Mr. Buchanan's own words this melo-dramatic scene was thus enacted. Gillian (the wife of the "dead" O'Mara), her face flushed with free air and exercise, entered the hall, followed by Sir George, the accepted baronet, and stopped for a moment at the sight of the stranger. He, with his fingers still playing the melody, turned half round upon the music stool, "Gillian!" he said, softly smiling. . . . . "Philip!" She spoke the name scarcely louder than a whisper, and fell fainting in Sir George's arms. But the returned exile does not have it all his own way—after this—Nemesis is already awaiting him, and later on in the book we find Mr. O'Mara with an avenging bowie knife through his heart, placed there by the unforgiving hand of a certain Jake, who had himself been previously injured by this hero of the book.

Dr. Janet of Harley Street. By Dr. Arabella Kenealy. (London: Digby, Long, and Co., 1893.)

Dr. Arabella Kenealy writes this book with a definite knowledge of her subject, being a member of the medical profession herself, and having passed through the varied phases of practical experience contingent thereto. The plot of the story, which is based on somewhat new and original lines, maintains its interest throughout, the dialogues are bright and clever, the style easy. The strongest personality in these pages is undoubtedly Dr. Janet herself, whose presence

**THE BOOK WORLD FOR WOMEN AND NURSES**—*continued.*

throughout is the embodiment of invigorating energy, which one can almost say is infectious. She presents an interesting study of a woman doctor, having lost none of the tender sympathy of a woman's nature, in the cultivation of a strongly masculine intellect. To her in certain social difficulties comes the second heroine in the book, Janet de Richeville, a young and inexperienced girl, who, when little more than a child, is thrown, or rather throws herself, upon the world's mercy in search of a livelihood. Dr. Janet befriends and protects the girl, superintending her *protégé's* training preparatory to her swelling the ranks of female practitioners. Throughout this part of the narrative the dramatic force of the book is further enhanced by love complications, and a romance is deftly interwoven, a romance which culminates in rescuing the girl from a career to which nature had ill designed her. A delicately strong, highly sensitive plant, who saw science through the medium of sentiment, "yet," observes the writer, "she would have found her tasks easier could she have thrown off the control of her woman nature, and have worked with a dispassionate mind. For example, in the study of botany, it was always a difficulty to her to see a leaf or flower from its purely 'lanceolate,' petiolate or pinnatified standpoints; like a subtle shallowy wraith, the fading dissected blossom clung about her sense, telling her truths that science left untouched." But in truth it would have taken a lifetime of mental plane-shifting to have displaced Miss Janet's emotional impulses by intellectual interests, and one heaves a sigh of relief when the young girl's mission descends to woman's usual fate. But the interest of the book is centred round Dr. Janet, whose personality is realised so vividly that one regrets on putting down the book one has not seen more of her.

### REVIEWS OF THE MONTH.

The Humanitarian for December contains an article on "The Taxation of Pleasure." In a word, the writer suggests that the public should pay a few extra pennies for every entertainment they attend, which should form a fund for old age pensions. He would not suggest a tax on commodities, but that theatres form a legitimate and reasonable field from which to reap from the rich to help the poor. The tax in the form of a stamp would be applied as a percentage on the price of the ticket, so that the gathering should fall chiefly on the well-to-do. It is not proposed to carry out the system in respect to entertainments which combine instruction with amusement, such as the Zoological Gardens, the Tower, &c. Mr. Holyoake's scheme has been considered by a good many, and doubtless it is a form of taxation which will appeal to the interest of so many that a warm discussion cannot fail to ensue. Sir Dyce Duckworth contributes a most fair and impartial criticism on the use and abuse of alcohol. The strenuous advocate of teetotalism and the abusers of the vine will alike find much to ponder over in these pages. Like all thoughtful men, and those who have watched results, Sir Dyce Duckworth points out that it is not to legislature we must look to free our nation from the curse of drink, but to the steady, restraining influences of civilising education and to the teaching of Christianity.

